# Comparing the neural distance effect derived from the non–symbolic comparison and the same–different task

**DOI:** 10.3389/fnhum.2013.00028

**Published:** 2013-02-14

**Authors:** Karolien Smets, Titia Gebuis, Bert Reynvoet

**Affiliations:** ^1^Laboratory of Experimental Psychology, Faculty of Psychology and Educational Sciences, University of LeuvenLeuven, Belgium; ^2^Subfaculty of Psychology and Educational Sciences, University of LeuvenKortrijk, Belgium

**Keywords:** number processing, comparison, same–different, non–symbolic, distance effect, EEG

## Abstract

As a result of the representation of numerosities, more accurate and faster discrimination between two numerosities is observed when the distance between them increases. In previous studies, the comparison and same-different task were most frequently used to investigate this distance effect. Recently, it was questioned whether the non-symbolic distance effects derived from these tasks originate at the same level. In the current study, we examined the behavioral and neural distance effects of the comparison and same-different task to assess potential differences between both tasks. Participants were first year university students. Each participant completed both tasks, while their reaction time, accuracy and brain activity on predefined components was measured. The early N1-P2p transition and the P2p component on temporo-occipital (TO) and inferior parietal (IP) electrode groups were considered, as well as the late P3 component on a central (C) electrode group. The results showed that the behavioral distance effects from both tasks were comparable, although participants' performance was worse on the same-different task. The neural results revealed similar effects of distance on the mean amplitudes for the early components for both tasks (all *p*′s < 0.02) and an additional effect of task difficulty on the mean amplitudes of these components. Similar as in previous studies, we found a (marginally) significant increase in mean amplitude of the later P3 component with increasing distance for the comparison (*p* = 0.07), but not for the same-different task. Apparently, the initial stages of number processing are comparable for both tasks, but an additional later stage is only present for the comparison task. The P3 effect would be indicative of this decisional stage, which was previously proposed to underlie the comparison distance effect (CDE).

## Introduction

The non-symbolic comparison distance effect (CDE) refers to the more accurate and faster discrimination between two numerosities that are farther apart (e.g., 2 dots and 10 dots) than between two numerosities that are closer to each other (e.g., 2 dots and 4 dots). This effect is obtained when participants have to indicate the larger of those numerosities (i.e., the comparison task) and is present at different ages (e.g., Buckley and Gillman, 1974; Sasanguie et al., 2012). Although a large number of behavioral and neuroimaging studies investigated the non-symbolic CDE, the discussion regarding its origin is not yet resolved. There are two main theories about the source of the non-symbolic CDE: the representational overlap view and the response-related or decisional mechanisms view.

The *representational overlap view* is the commonly held view that the CDE arises because of overlap in the magnitude representations (e.g., Restle, 1970; Libertus and Brannon, 2010). More specifically, the activation pattern of each numerosity is a Gaussian distribution that peaks at the target numerosity and decreases with increasing distance from the target numerosity. As a result, numerosities that are numerically closer to each other will have more representational overlap than numerosities that are numerically farther apart. The ability to discriminate between two numerosities therefore depends on the amount of representational overlap between the numerosities that need to be compared. A larger overlap leads to a lower accuracy rate and a longer reaction time (i.e., the distance effect).

In contrast, the *response-related or decisional mechanisms* view (e.g., Dehaene, 1996; Göbel and Rushworth, 2004; Shaki et al., 2006) states that the numerical distance effect can also be explained by a difference in weights between the relevant magnitudes and the response categories. This idea was underlined by the modeling data of Verguts et al. (2005). In line with this reasoning, Cohen Kadosh et al. (2008) suggested that the CDE is caused by a mechanism that is independent of mental representations of the manipulated features, but instead is affected by stimulus saliency (e.g., response selection). This mechanism is suggested to be specific to tasks where stimuli have to be explicitly compared by indicating “more” or “less,” such as the comparison task.

Van Opstal and Verguts (2011) attempted to disentangle the representational overlap view and the decisional mechanisms view by investigating number reasoning with an alternative task: the same-different task (e.g., Dehaene and Akhavein, 1995; Gebuis and Van der Smagt, 2011; Sasanguie et al., 2011). Here, participants are presented with two numerosities and have to indicate whether both represent the same or a different numerosity. Similar as for comparison, participants are more accurate and faster when the distance between the two numerosities increases, an effect we will refer to as the same-different distance effect (SDDE). Van Opstal and Verguts (2011) found in their model simulations as well as in their behavioral experiments, that the SDDE is caused by the broad tuning curves of the numerosities which is directly related to the underlying stimulus representations. Hence, the SDDE can only be accounted for by assuming representational overlap between close numerosities (i.e., the representational overlap view) and not by decisional mechanisms. Potential differences between the CDE and the SDDE are therefore linked to differences in the origin of both distance effects.

Cohen Kadosh et al. (2008) provided evidence in favor of the decisional mechanisms view as the origin for the CDE but not the SDDE. They examined the presence of a CDE and SDDE using pitch stimuli (low and high music pitch discrimination). Their results showed a significant CDE, but no SDDE. The fact that they found a CDE is already surprising given the clear differences between the mental representation of pitch (two-dimensional) and other magnitudes (one-dimensional). If the CDE in pitch was caused by overlap in the mental representations of pitch stimuli, this overlap should also lead to a SDDE according to the representational overlap view. However, a SDDE was absent in their data, implicating a dissociation between the CDE and the SDDE. Cohen Kadosh et al. (2008) concluded that decisional mechanisms instead of representational overlap induced the CDE in their study. In contrast, the SDDE can only be accounted for by representational overlap, which is not present for pitch. Together, these results point in the direction of a different origin for both distance effects.

Differences between the CDE and the SDDE were also found with respect to their developmental pattern. The developmental pattern of the CDE and the SDDE should be the same if both distance effects are supported by the same neural mechanisms. However, the CDE was found to decrease with increasing age (e.g., Holloway and Ansari, 2008; Sasanguie et al., 2011), while this was not observed for the SDDE (Duncan and McFarland, 1980; Defever et al., 2012). Nevertheless, a significant relationship between both distance effects was also found, indicating that both effects may still be related (Sasanguie et al., 2012).

To date, there is no consensus about the origin of the non-symbolic distance effects obtained with the comparison and same-different task. It is often implicitly assumed that the same neural mechanisms give rise to the CDE and the SDDE. A way to provide more insight in the mechanisms underlying the distance effects from these different tasks is by evaluating their underlying neural mechanisms using electroencephalography (EEG). Several studies already examined event-related potentials (ERPs) of the CDE. For instance, Libertus et al. (2007) showed that the CDE of non-symbolic number stimuli affected the transition between the N1 and the P2p component and the P2p component itself at temporo-occipital (TO) and inferior parietal (IP) electrode groups. The CDE also affected the P3 component at central (C) electrode groups. This P3 component was suggested to relate to decisional instead of numerosity processes. However, the absence of neural data of the SDDE does not enable a direct comparison of the neural distance effects of the comparison and the same-different task.

The current research therefore focuses on the neural mechanisms of the non-symbolic distance effects derived from both the comparison and same-different task, using EEG as a measure. According to the representational overlap view, both distance effects arise because of overlap in neural activation of the numerosities. Hence, the same neural mechanisms should underlie both distance effects and the neural distance effects should be similar. On the contrary, if the CDE is supported by decisional mechanisms and the SDDE by representational overlap between close numerosities, it is expected that both distance effects have a different time course and/or that the neural distance effect is present on different electrode groups in the different tasks. The potential differences between the comparison and the same-different task are most likely to arise in the later stages of processing, which are more related to decisional or response processes.

## Materials and methods

### Participants

Twenty-four normal university students participated in the study of which 17 were included in the final analyses. Four participants were excluded from the analyses because of a measurement error at the time of testing. Another three participants were excluded, because more than 25% of their trials contained artifacts in the EEG signal in either one of the tasks (see below). The final sample consisted of 14 women and 3 men with a mean age of 20 years (*SD* = 1.60). Participants were either paid for their participation or received course credits. All participants gave written informed consent for their participation. The experiment was approved by the Ethical Committee of the Faculty of Psychology and Educational Sciences of the University of Leuven.

### Apparatus

The stimuli were presented on a 17-inch color screen. The presentation of the stimuli and recording of the behavioral data was controlled by MatLab 7.1, using the Psychophysics Toolbox.

### Stimuli

Two solid gray circles were presented on a black background. One circle was presented on the left of the screen and one on the right of the screen. The circles were 2.3° visual angle in diameter and each circle contained a dot pattern. We created these dot patterns with an adapted version of the program developed by Gebuis and Reynvoet (2011a) (available at http://titiagebuis.eu/Materials.html). This script creates stimuli where the more numerous stimulus has visual cues that are larger in half of the trials and smaller in the other half of the trials. Hence, a single visual cue is not informative about number. Furthermore, the program varies the relevant visual cues (dot diameter, convex hull, contour length, aggregate surface, and density) in such a manner that the difference in visual properties does not correlate with the number distance between the two presented stimuli throughout the experiment. *Post hoc* analyses on the stimuli included in the analyses confirmed this notion: the average diameter of the dots (comparison: *R*^2^ < 0.01, *SD* < 0.01; same-different: *R*^2^ < 0.01, *SD* < 0.01), convex hull (comparison: *R*^2^ = 0.11, *SD* = 0.04; same-different: *R*^2^ = 0.07, *SD* = 0.03), contour length (comparison: *R*^2^ = 0.01, *SD* < 0.01; same-different: *R*^2^ = 0.01, *SD* < 0.01), aggregate surface (comparison: *R*^2^ < 0.01, *SD* < 0.01; same-different: *R*^2^ = 0.01, *SD* < 0.01), and density (comparison: *R*^2^ < 0.01, *SD* < 0.01; same-different: *R*^2^ < 0.01, *SD* < 0.01) could only explain a negligible amount of the total variance in number distance.

We presented participants with two tasks: a non-symbolic comparison task and a non-symbolic same-different task. In the comparison task, we manipulated the distance between the numerosities, resulting in three distance conditions: a distance of one unit (dot arrays: 1-2, 2-1, 3-4, 4-3, 7-6, 6-7, 8-9, 9-8), a distance of two units (dot arrays: 2-4, 4-2, 3-1, 1-3, 7-9, 9-7, 8-6, 6-8), and a distance of three units (dot arrays: 1-4, 4-1, 6-9, 9-6). Each number pair was presented 6 times for distances 1 and 2 and 12 times for distance 3. This was done to obtain a balanced set where each condition consisted of an equal number of trials. For the same-different task, half of the trials were the same numerosity and the other half differed in numerosity. For the “different” trials the same number pairs as for the comparison task were used. Eight “same” trials were added (dot arrays: 1-1, 2-2, 3-3, 4-4, 6-6, 7-7, 8-8, 9-9) and they were each presented 18 times throughout the task. This was done to obtain 50% “same” trials and 50% “different” trials. Thus, the comparison task consisted of 144 trials in total (48 per distance), while the same-different task consisted of 144 “different” trials and 144 “same” trials (288 in total).

### Procedure

The participants completed both the non-symbolic comparison task and the non-symbolic same-different task. The order of these tasks was randomized. For the comparison task, participants were instructed to indicate the larger of the two numerosities by pressing the corresponding key (left key for left number larger and right key for right number larger). For the same-different task, participants had to press left if they thought both numerosities were the same in numerosity and right if they thought they differed in numerosity. Participants were required to respond as quickly and as accurately as possible.

**Figure 1:**
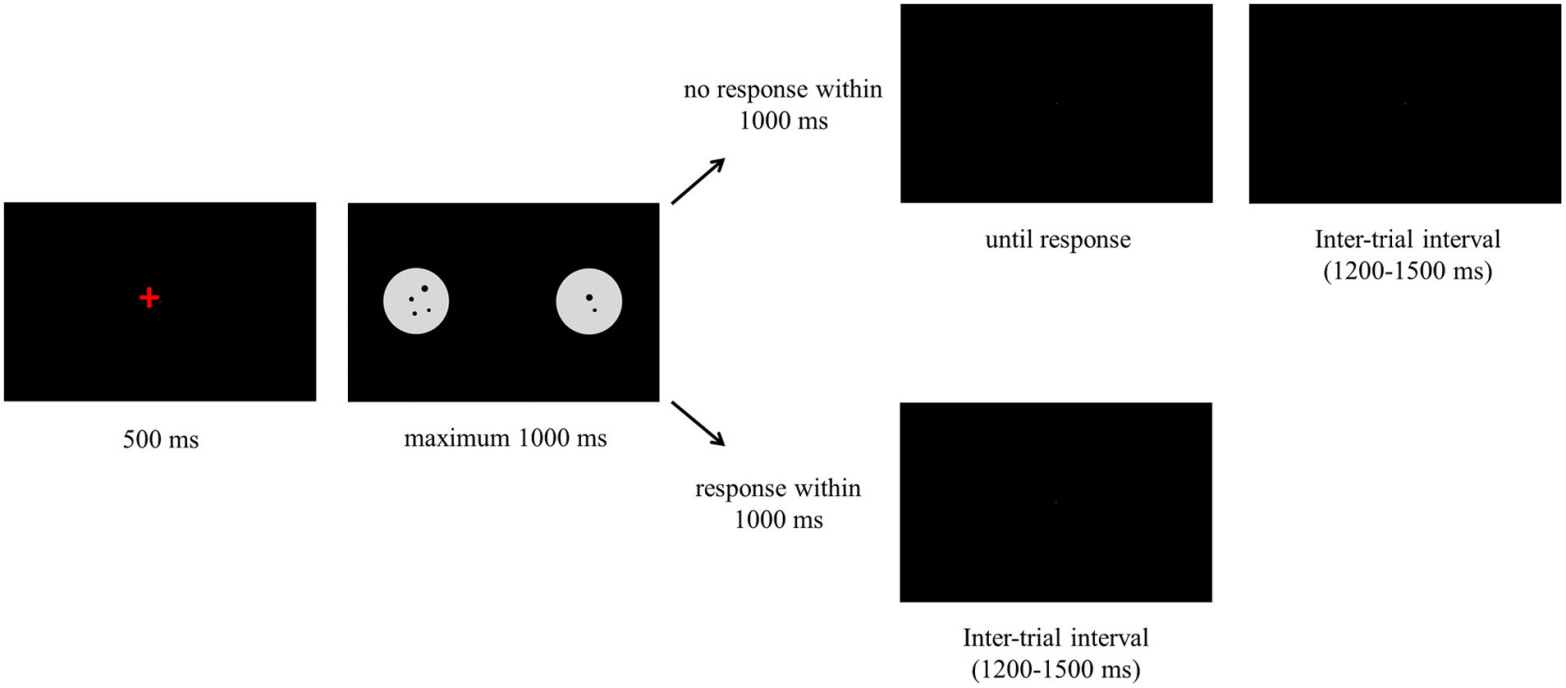
**Presentation of the stimuli in one trial for the comparison and same-different task**.

Each trial started with a fixation cross that was presented for 500 ms. Next, the stimulus was presented and it remained on the screen for 1000 ms. Participants could either respond during the time the stimulus was on the screen or after it disappeared. If participants responded when the stimulus was on the screen, the next trial started immediately. If participants did not respond when the stimulus was on the screen, a black screen was displayed until a response was registered. The inter-trial interval varied between 1200 and 1500 ms. We included one break in the comparison task and three breaks in the same-different task. Before each task started, participants were presented with 15 practice trials. The procedure of both tasks is illustrated in Figure 1 and is the same for the comparison and same-different task. The tasks only differed in the instruction that was given to the participants.

### Electrophysiological recordings

Brain activity was recorded from 64 electrodes according to the International 10/20 EEG system (with a sampling rate of 2048 Hz) by means of the Active Two System (BioSemi, Amsterdam, The Netherlands, for an explanation see http://www.biosemi.com). The horizontal electro-oculogram (EOG) was recorded from the outer canthi of both eyes and the vertical EOG was recorded from two electrodes, one attached above and the other below the left eye.

### ERP preprocessing

The data were analyzed using EEGLAB in MatLab. The EEG signals were off-line re-referenced to the average of all 64 electrodes and noisy electrodes were interpolated using spherical splines. The EEG signal was first corrected for eye movements using independent component analysis (ICA). Next, it was filtered with a 40 Hz low pass filter. The data were divided into epochs from 200 ms before stimulus presentation until 1200 ms after. For baseline correction, we used the time window from –200 to 0 ms. Trials with an incorrect response or a response time longer than 1200 ms and trials with artifacts (maximum or minimum of ±100 μV) were rejected from the ERP analyses. In accordance with previous research (e.g., Sasanguie et al., 2011), only the “different” trials of the same-different task were included in the ERP analyses. When more than 25% of the trials of a participant still contained artifacts in the EEG signal after correction for eye movements in either one of the two tasks, the participant was discarded from the ERP and behavioral analyses. This resulted in the exclusion of three participants.

### Behavioral analyses

Trials with a reaction time longer than 1200 ms and trials that contained artifacts in the EEG signal were rejected from further behavioral analyses to have the same set of trials in both the ERP and behavioral analyses. For the same-different task, only the “different” trials were included.

Mean accuracies for all responses and median reaction times for the correct responses were calculated for both tasks, for each number distance and for each participant. For accuracy and reaction time separately, we conducted a repeated measures analysis with task (two levels: comparison and same-different) and distance (three levels: distances 1–3) as within-subjects factors. When the assumption of sphericity was violated in the analyses, we corrected the *p*-values with the Greenhouse–Geisser correction (pGG).

### Electrophysiological analyses

We calculated grand average ERPs for each distance condition on the same three electrode groups as in the study of Libertus et al. (2007): TO (left electrodes: TP7, P7, P9, PO7; right electrodes: TP8, P8, P10, PO9), IP (left electrodes: PO3, PO7, O1; right electrodes: PO4, PO8, O2), and C (left electrodes: CP1, CP3, CP5, P1, P3, P5, P7; right electrodes: CP2, CP4, CP6, P2, P4, P6, P8). At the TO and IP electrodes groups, we investigated the transition between the N1 component and the P2p component, and the P2p component. At the C electrode groups, we examined the P3 component. The following time windows were used: N1-P2p: 220–256 ms, P2p: 280–344 ms, and P3: 380–470 ms. These time windows were partly based on the time windows in the study of Libertus et al. (2007) and partly on visual inspection of the data (the peaks of the N1, P2p, and P3 components). Both the electrode groups as the time windows are in correspondence with previous research (e.g., Dehaene, 1996; Temple and Posner, 1998; Libertus et al., 2007).

For each component and for each electrode group, we conducted a repeated measures analysis with task (two levels: comparison and same-different), hemisphere (two levels: left and right hemisphere), and distance (three levels: distances 1–3) as within-subjects factors. We used the mean amplitudes of the time windows of these components as dependent variables in these analyses. If necessary, *p*-values were corrected with the Greenhouse–Geisser correction (pGG).

## Results

### Behavioral results

**Figure 2:**
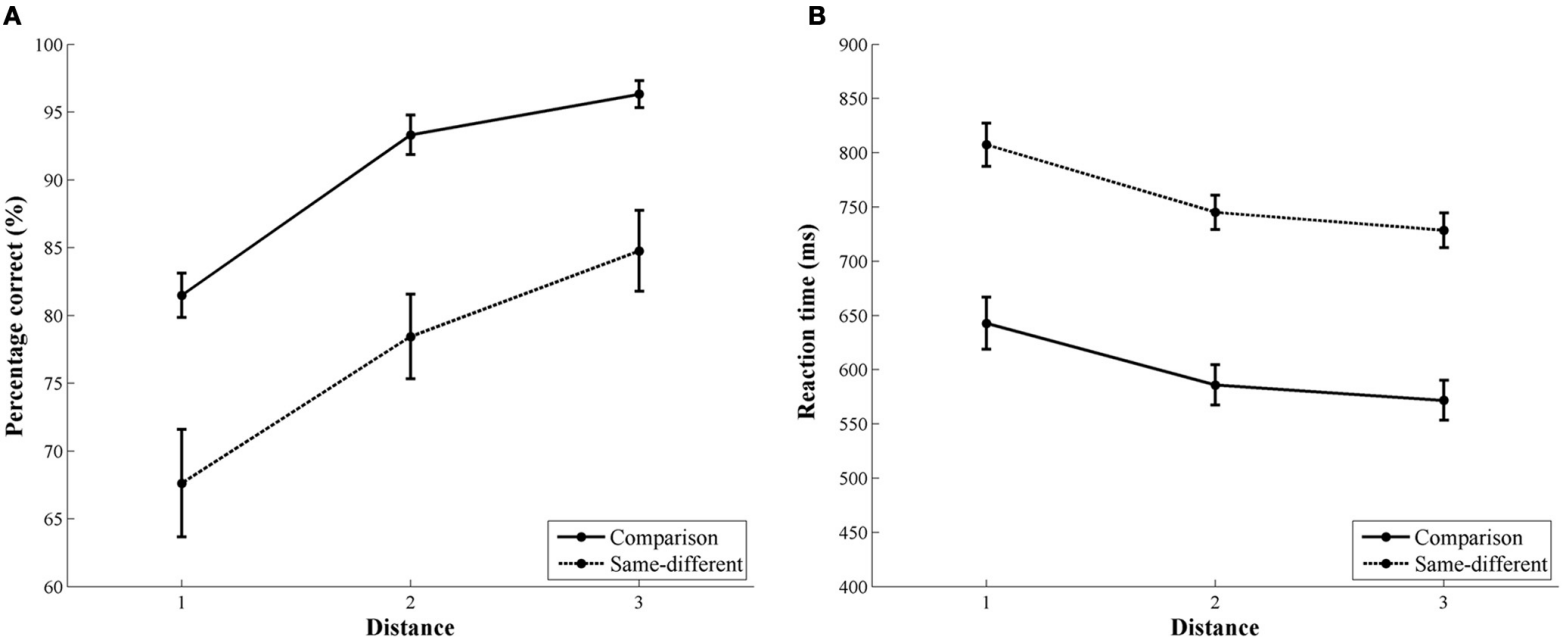
**Behavioral results of the comparison and the same-different task.** The left image **(A)** represents the accuracy rate for the comparison (solid line) and the same-different task (dotted line). The right image **(B)** represents the reaction time data for the comparison (solid line) and the same-different task (dotted line). For both tasks, a distance effect was present in accuracy and reaction time.

For accuracy, we found a significant main effect of task [*F*_(1, 32)_ = 33.19, *p* < 0.001]. Participants were more accurate in the comparison task (90%) than in the same-different task (77%). The main effect of distance was also significant [*F*_(2, 32)_ = 77.08, *p* < 0.001]. Linear contrasts indicated that accuracy increased with increasing number distance [*F*_(1, 16)_ = 184.61, *p* < 0.001; distance 1: 75%, distance 2: 86%, and distance 3: 91%]. The interaction between task and distance did not reach significance [*F*_(2, 32)_ = 0.55, *p* = 0.59], indicating no difference in the distance effects of both tasks. In Figure 2A, the distance effect in accuracy of the comparison and same-different task is illustrated.

The results for reaction time were similar to those of the accuracy data. The main effect of task was significant [*F*_(1, 16)_ = 87.15, *p* < 0.001]. Participants were faster in the comparison task (600 ms) than in the same-different task (760 ms). A significant main effect of distance was also present [*F*_(2, 32)_ = 44.90, *pGG* < 0.001]. Linear contrasts showed that reaction times decreased with increasing number distance [*F*_(1, 16)_ = 56.24, *p* < 0.001; distance 1 = 725 ms, distance 2 = 666 ms, and distance 3 = 650 ms]. The interaction between task and distance was not significant [*F*_(2, 32)_ = 0.17, *p* = 0.85]. The distance effect in reaction time for the comparison and the same-different task is shown in Figure 2B.

### Electrophysiological results

#### Temporo-occipital electrode group

**Figure 3:**
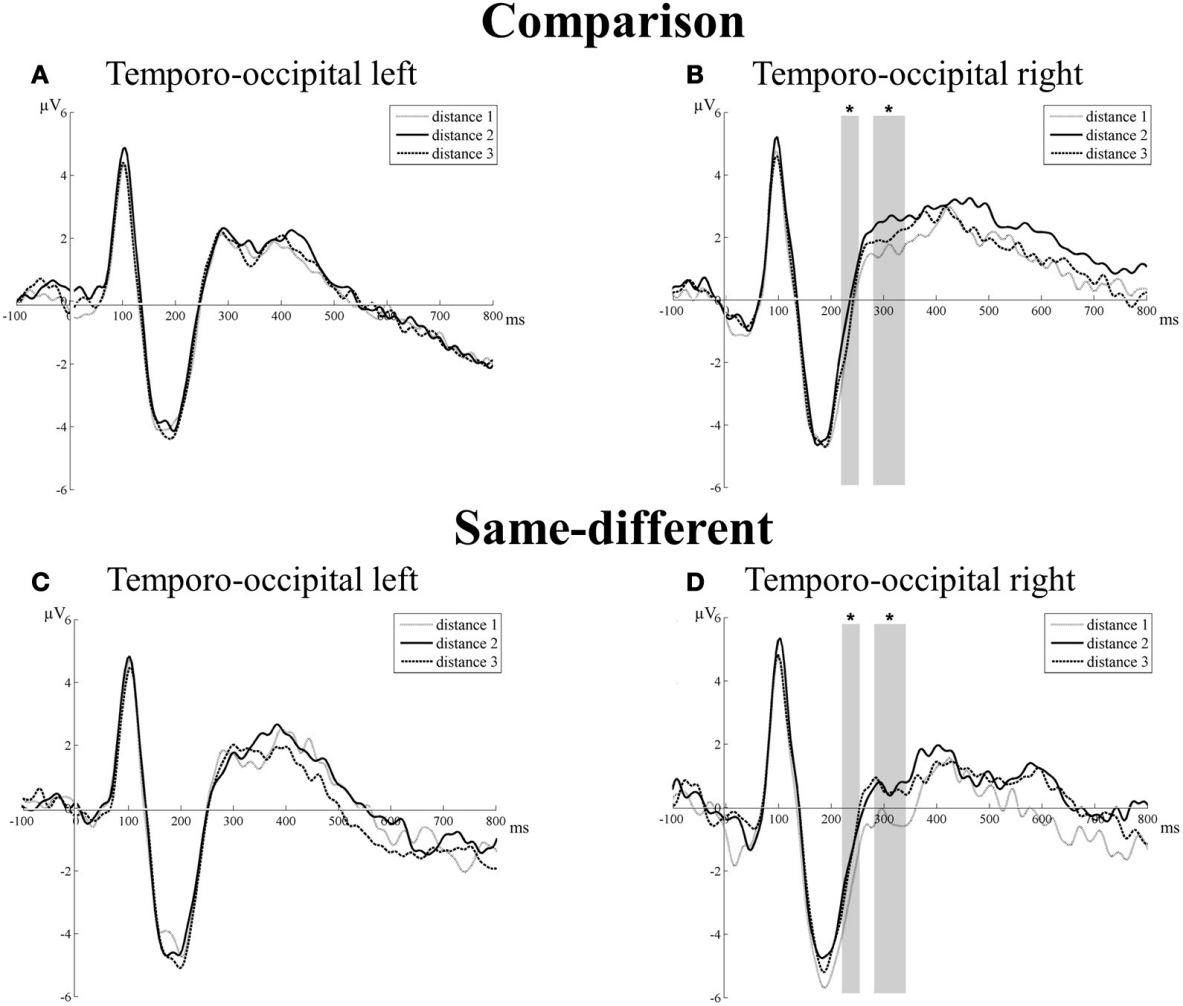
**Neural results for the temporo-occipital electrode group of the comparison (A and B) and the same-different task (C and D) for the left and right hemisphere.** Significant effects of distance are indicated with an asterisk. A distance effect was present in the comparison and same-different task for the transition from the N1 component to the P2p component (220–256 ms) and for the P2p component (280–344 ms), but only in the right hemisphere. This is illustrated in **(B)** for the comparison task and in **(D)** for the same-different task.

***Transition from N1 component to P2p component.*** There was a significant main effect of task [*F*_(1, 16)_ = 8.33, *p* = 0.01]. The mean amplitude of the same-different task was more negative than the mean amplitude of the comparison task (–2.19 μV vs. –0.99 μV, respectively). The main effect of hemisphere did not reach significance [*F*_(1, 16)_ = 0.07, *p* = 0.80]. The main effect of distance was also not significant [*F*_(2, 32)_ = 2.63, *p* = 0.09], but distance interacted with hemisphere [*F*_(2, 32)_ = 4.35, *p* = 0.02]. This interaction was the result of a gradual increase in mean amplitudes with increasing distance in the right, but not in the left hemisphere. Linear contrasts of the mean amplitudes of the different distances in the right hemisphere indicated the presence of an increase in mean amplitude with increasing distance [*F*_(1, 16)_ = 6.48, *p* = 0.02]. Pairwise *t*-tests were conducted to further unravel this effect and indicated a significant difference in mean amplitude in the right hemisphere over both tasks between distance 1 and distance 2 [*t*_(16)_ = 3.15, *p* = 0.006], between distance 1 and distance 3 [*t*_(16)_ = 2.55, *p* = 0.02], but not between distance 2 and distance 3 [*t*_(16)_ = 1.48, *p* = 0.16]. In contrast, linear contrasts in the left hemisphere were not significant [*F*_(1, 16)_ = 0.11, *p* = 0.92]. The other two-way interaction effects (all *F*′s < 2.57 and all *p*′s > 0.13) and the three-way interaction between task, hemisphere, and distance did not reach significance [*F*_(2, 32)_ = 0.79, *p* = 0.46]. Thus, we did not find differences between the distance effects of the comparison and the same-different task. For both tasks, a distance effect was present at the right TO electrode group between 220 and 256 ms (see Figures 3B,D), but not at the left hemisphere (see Figures 3A,C). Additionally, there was also a difference in mean amplitude between both tasks.

***P2p component.*** For the P2p component, there was a significant main effect of task [*F*_(1, 16)_ = 6.64, *p* = 0.02]: the mean amplitude of the comparison task was more positive than the mean amplitude of the same-different task (1.95 and 0.99 μV, respectively). The main effect of hemisphere was not significant [*F*_(1, 16)_ = 0.73, *p* = 0.41], but there was a significant interaction between task and hemisphere [*F*_(1, 16)_ = 6.58, *p* = 0.02]. Pairwise *t*-tests did not reveal a significant difference in mean amplitude between tasks for the left hemisphere [*t*_(16)_ = 0.44, *p* = 0.67], but the difference in mean amplitude between the comparison and same-different task was significant for the right hemisphere [*t*_(16)_ = 3.44, *p* = 0.003]. The mean amplitude in the right hemisphere across the different distances of the same-different task was significantly smaller than the mean amplitude of the comparison task. Furthermore, the main effect of distance was significant [*F*_(2, 32)_ = 4.29, *p* = 0.022] and distance interacted with hemisphere [*F*_(2, 32)_ = 4.86, *p* = 0.01]. This interaction resulted from an increase in amplitude with increasing number distance in the right hemisphere, but not in the left hemisphere. Linear contrast analyses of the mean amplitudes per distance showed a significant increase in mean amplitude with increasing distance in the right hemisphere [*F*_(1, 16)_ = 9.29, *p* = 0.008], which was not present in the left hemisphere [*F*_(1, 16)_ = 0.02, *p* = 0.73]. Pairwise *t*-tests on the mean amplitudes in the right hemisphere over both tasks showed a significant difference between distance 1 and distance 2 [*t*_(16)_ = 4.31, *p* = 0.001], between distance 1 and distance 3 [*t*_(16)_ = 3.05, *p* = 0.008], but not between distance 2 and distance 3 [*t*_(16)_ = 0.97, *p* = 0.35]. The interaction between task and distance [*F*_(2, 32)_ = 0.40, *p* = 0.67] and the three-way interaction between task, hemisphere, and distance did not reach significance [*F*_(2, 32)_ = 0.03, *p* = 0.97]. Thus, similar as for the transition from the N1 to the P2p component, a distance effect was present at the P2p component in the right hemisphere of the TO electrode group. There were again no differences in the distance effects of both tasks. There was however a significant difference in mean amplitude of the right hemisphere between both tasks. The results of the TO electrode group are illustrated in Figures 3A,C for the left hemisphere and in Figures 3B,D for the right hemisphere.

#### Inferior parietal electrode group

**Figure 4:**
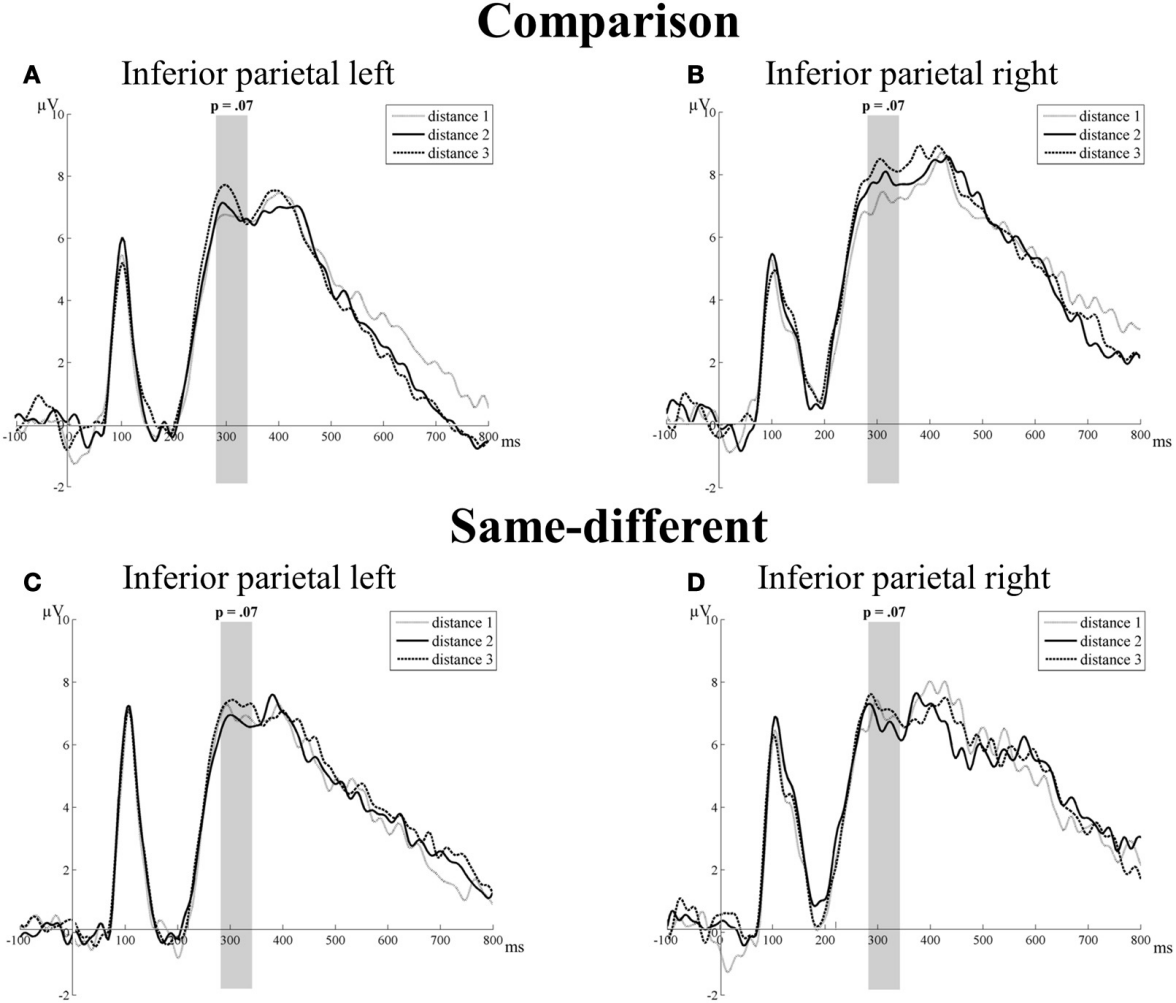
**Neural results for the inferior parietal electrode group of the comparison (A and B) and the same-different task (C and D) for the left and right hemisphere.** Marginally significant distance effects are indicated with the corresponding *p*-value. A trend toward a distance effect was present for the comparison and same-different task for the P2p component (280–344 ms). This is illustrated in **(A)** and **(B)** for the comparison task and in **(C)** and **(D)** for the same-different task.

***Transition from the N1 component to the P2p component.*** The main effect of task [*F*_(1, 16)_ = 3.13, *p* = 0.10] and distance were not significant [*F*_(2, 32)_ = 1.49, *p* = 0.24]. Only the main effect of hemisphere reached significance [*F*_(1, 16)_ = 5.45, *p* = 0.03]: the mean amplitude of the left hemisphere was smaller than the mean amplitude of the right hemisphere (2.31 vs. 4.13 μV, respectively). The other two-way interactions (all *F*′s < 0.71 and *p*′s > 0.41) and the three-way interaction between task, hemisphere, and distance were not significant [*F*_(2, 32)_ = 0.23, *p* = 0.79]. Thus, we did not find significant effects of distance for the transition from the N1 component to the P2p component at the IP electrode group for the comparison and the same-different task (see Figure 4).

***P2p component.*** There was no significant main effect of task [*F*_(1, 16)_ = 0.69, *p* = 0.42] or hemisphere [*F*_(1, 16)_ = 0.24, *p* = 0.63]. The main effect of distance showed a trend toward significance [*F*_(2, 32)_ = 2.92, *p* = 0.07]. Linear contrasts showed that this trend was the result of a significant increase with increasing distance [*F*_(1, 16)_ = 7.87, *p* = 0.01]. Pairwise *t*-tests showed a significant difference between distance 1 and distance 3 [*t*_(16)_ = 2.81, *p* = 0.01] and a marginally significant difference between distance 2 and distance 3 [*t*_(16)_ = 1.95, *p* = 0.07]. The difference between distance 1 and distance 2 was not significant [*t*_(16)_ = 0.29, *p* = 0.78]. All two-way interactions (all *F*′s < 2.79 and *p*′s > 0.11) and the three-way interaction between task, hemisphere, and distance were not significant [*F*_(2, 32)_ = 0.42, *p* = 0.66]. Thus, a trend toward an effect of distance was present for the P2p component at the IP electrode group in both tasks. There were no significant differences between the distance effects of the comparison and the same-different task. This is illustrated in Figure 4.

#### Central electrode group

**Figure 5:**
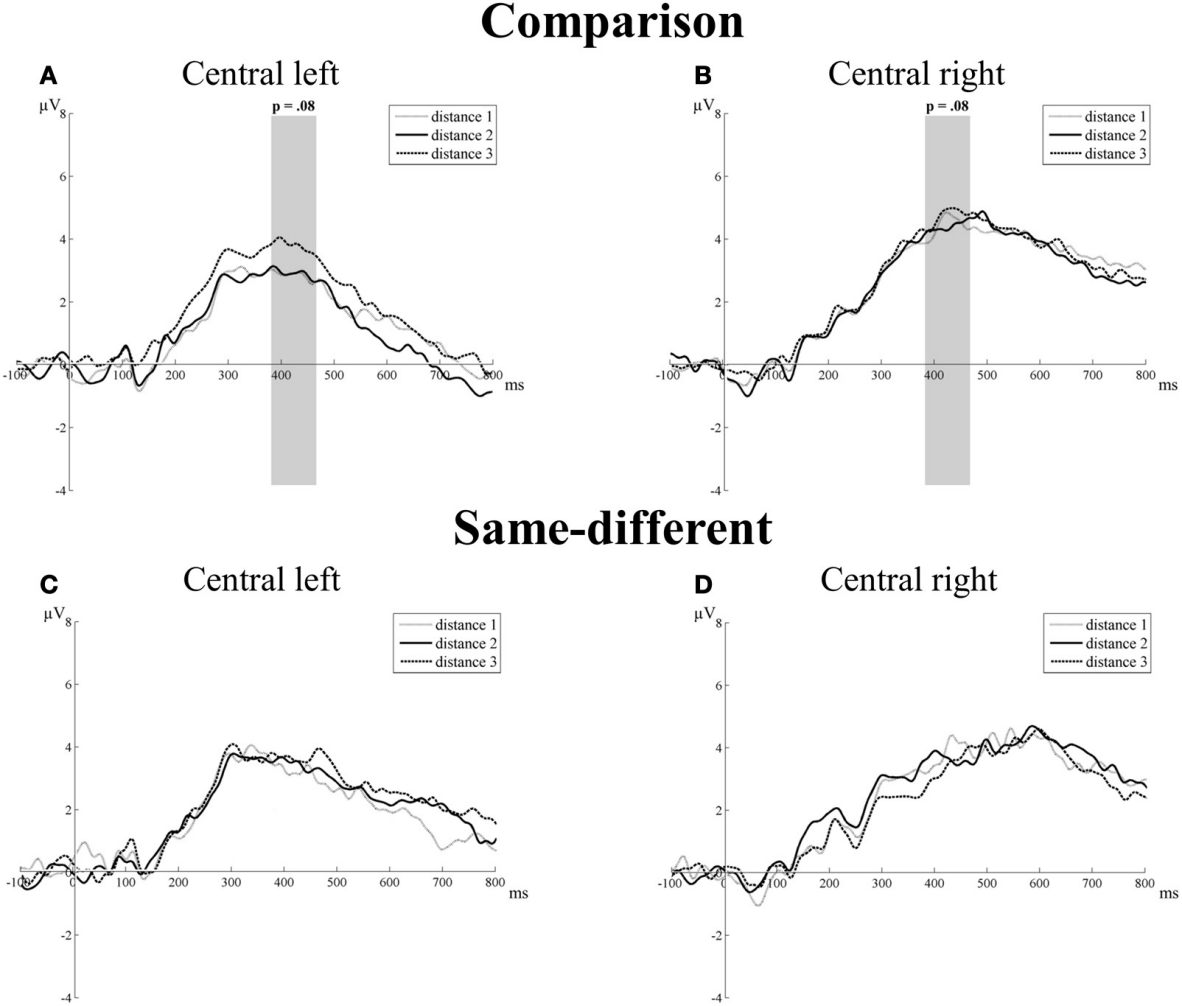
**Neural results for central electrode group of the comparison (A and B) and the same-different task (C and D) for the left and right hemisphere.** Marginally significant distance effects are indicated with the corresponding *p*-value. A trend toward a distance effect was present for the P3 component (380–470 ms), but only in the comparison task. This is illustrated in **(A)** for the left hemisphere of the comparison task and in **(B)** for the right hemisphere of the comparison task.

***P3 component.*** There was no significant main effect of task [*F*_(1, 16)_ = 0.80, *p* = 0.38], hemisphere [*F*_(1, 16)_ = 1.23, *p* = 0.29], or distance [*F*_(2, 32)_ = 1.66, *pGG* = 0.21]. The interaction between task and hemisphere reached significance [*F*_(1, 16)_ = 13.04, *p* = 0.002]. This was due to differences in the mean amplitudes between the left (3.11 μV) and the right hemisphere (4.47 μV) in the comparison task [*t*_(16)_ = 2.09, *p* = 0.05], but not in the same-different task [*t*_(16)_ = 0.18, *p* = 0.86]. The interaction between task and distance showed a trend toward significance [*F*_(2, 32)_ = 2.59, *p* = 0.08]. A closer inspection of the data showed that this trend resulted from an increase in the mean amplitudes with increasing number distance for the comparison task (distance 1 = 3.57, distance 2 = 3.59, and distance 3 = 4.21), but not for the same-different task (distance 1 = 3.46, distance 2 = 3.49, and distance 3 = 3.47). Linear contrasts showed a marginally significant trend in function of distance [*F*_(1, 16)_ = 3.84, *p* = 0.07] for the comparison task, which was not present for the same-different task [*F*_(1, 16)_ = 0.003, *p* = 0.96]. The interaction between hemisphere and distance was not significant [*F*_(2, 32)_ = 2.14, *p* = 0.14], as was the three-way interaction between task, hemisphere, and distance [*F*_(2, 32)_ = 0.35, *p* = 0.71]. Thus, the results point to an effect of distance for the P3 component on the C electrode group for the comparison task (Figures 5A,B), but not for the same-different task (Figures 5C,D).

## Discussion

It is debated whether the non-symbolic distance effects derived from the comparison and same-different task reflect the same neural processes (e.g., Verguts et al., 2005; Cohen Kadosh et al., 2008; Van Opstal et al., 2008). Behavioral studies regarding this issue were inconclusive. In the present study, we therefore investigated the neural mechanisms underlying both tasks. To this end, we presented participants with a non-symbolic comparison and a non-symbolic same-different task while measuring their brain activity with EEG.

The behavioral results showed a significant distance effect for accuracy and reaction time in both tasks. Participants were more accurate and faster in discriminating between numerosities that were farther apart than between numerosities that were closer to each other. This is in accordance with previous studies (Buckley and Gillman, 1974; Dehaene and Akhavein, 1995). In addition, we found that the same-different task was more difficult than the comparison task: participants performed worse and were slower to decide whether two numerosities represented the same or a different numerosity, which is also in correspondence with earlier studies (Piazza et al., 2004; Gebuis and Van der Smagt, 2011). In the present study, the difference in performance on both tasks could only result from the task itself (i.e., the task instruction: indicate which number is larger vs. indicate same or different), since all experimental parameters were similar in both tasks. Previous studies suggested that the task itself or the task instruction plays a role in a later processing stage, after visual or numerical processing is finished. This later stadium would support decisional- or response-related processes (Dehaene, 1996; Göbel and Rushworth, 2004; Verguts et al., 2005; Shaki et al., 2006). Irrespective of the similarity in the behavioral distance effects, the difference in performance between both tasks already puts forward that the CDE and the SDDE may result from different mechanisms. Considering that the SDDE can only be explained by the representational overlap view, the results therefore suggest that decisional mechanisms may be (partly) responsible for the CDE.

The ERP results showed an effect of distance at the right TO electrode group over both tasks. This effect was present from 220 to 344 ms at the transition from the N1 component to the P2p component and the P2p component itself. Overall, the mean amplitudes of these components increased with increasing number distance, although the difference in mean amplitude between distance 2 and distance 3 was not significant. The effect of distance seems to be limited to the right hemisphere. Previous studies found the right hemisphere to be more activated than the left hemisphere when adults process numerosities (e.g., Chochon et al., 1999; Piazza et al., 2002, 2007; Dormal and Pesenti, 2009; Cappelletti et al., 2010). This suggests that number characteristics such as the distance between the numerosities that need to be discriminated will have a stronger effect in this right hemisphere. However, other studies did not obtain this right lateralization in processing of non-symbolic numerosities (e.g., Pesenti et al., 2000; Cohen Kadosh et al., 2005; Cantlon et al., 2006), suggesting that the presence of the laterality in numerosity processing might be dependent on task-specific characteristics. As Holloway and Ansari (2010) already pointed out, this issue remains currently unresolved.

We also found a trend toward an increase in mean amplitudes with increasing number distance at the IP electrode group in both hemispheres and for both tasks. This effect was present at the P2p component between 280 and 344 ms for the comparison and the same-different task. Hence, the comparison and same-different task are similar with respect to the effect of distance on these early TO and IP components (N1-P2p and P2p component), which suggests that the first processing stages are similar in both tasks.

Although N1 and P2p amplitude effects were reported a number of times in the numerical cognition literature, controversy remains about what exactly these components reflect. Early amplitude effects in non-symbolic comparison were for instance attributed to number-specific processes (e.g., Temple and Posner, 1998; Piazza et al., 2002; Libertus et al., 2007; Hyde and Spelke, 2009), but also to differences in sensory properties of the number stimuli (Libertus et al., 2007; Gebuis and Reynvoet, 2012). The two explanations of these early components relate to different hypotheses in the numerical cognition literature about the neural mechanisms supporting numerosity processes. The number explanation suggests that numerosity processes are supported by the Approximate Number System that processes numerosity independent of its sensory properties (Cordes et al., 2001; Feigenson et al., 2004). In contrast, according to the sensory properties explanation, we rely on or are influenced by the sensory properties of the numerosity stimuli to judge number (Clearfield and Mix, 2001; Sophian and Chu, 2008; Dakin et al., 2011; Gebuis and Gevers, 2011; Gebuis and Reynvoet, 2011b).

It should be noted that our results show an increase in amplitude with increasing number distance for the TO and IP P2p component. This corresponds with the results of Experiment 1 in the study of Libertus et al. (2007). However, other researchers showed a decrease in amplitude of the P2p component with increasing distance (Hyde and Spelke, 2009; Experiment 2 of Temple and Posner, 1998; Libertus et al., 2007). Libertus et al. (2007) suggested that these opposite results are the outcome of differences in the sensory properties of the stimuli between the different studies (see also Gebuis and Reynvoet, 2012 for a similar reasoning). The early effects appear to reflect the processing of the sensory properties of the numerosity stimuli and the different control of sensory properties in different studies may be responsible for opposite results.

In addition to the similarities in the early stages of numerosity processing, differences were observed at a later stage. The P3 component (380–470 ms) at the C electrode group showed a marginally significant increase in mean amplitude with increasing number distance for the comparison, but not for the same-different task. Although the effect only shows a trend toward significance in the present study, the P3 effect for the comparison task is in line with previous studies (Nandrino and Massioui, 1995; Libertus et al., 2007; Gebuis et al., 2010), indicating good reason to assume it is present at least in the comparison task. However, what is evident and most important from the current study is that there is no sight of an effect of distance on the P3 component for the same-different task (*p* = 0.96).

Similar as for the early components, no consensus is reached about the mechanism underlying the P3 component. For instance, the P3 is suggested an index of attentional resources or working memory (Sutton et al., 1965; Polich and Kok, 1995; Gray et al., 2004), stimulus categorization and evaluation processes (comparison: larger or smaller; same-different: same or different) before the initiation of a response (Lansbergen and Kenemans, 2008; Gebuis et al., 2010), later stage response selection (Pritchard et al., 1999), and response processing (Donchin et al., 1978, 1986; Nandrino and Massioui, 1995; Cohen Kadosh et al., 2007; Libertus et al., 2007). Together, previous studies suggest that the origin of the P3 effect is more related to general cognitive abilities or later stages of stimulus processing (e.g., response categorization, preparation, selection, or initiation).

Next to the effects of distance on the mean amplitudes of the TO and IP electrode groups, a task difficulty effect was also present. On the TO electrode groups, the interaction between task and hemisphere indicated that the mean amplitude across distances of the same-different task was significantly smaller than that of the comparison task. Thus, the more difficult task as also indicated by the behavioral results is characterized by a smaller mean amplitude. This effect is along the same lines as the effect of distance in the present study: trials with smaller distance between the numerosities, which makes them more difficult for the participants to discern, are characterized by smaller mean amplitude. Moreover, this task-related effect reveals itself in the exact same right TO hemisphere where the effect of distance is also present. Similar as mentioned above, task difficulty in the current study could only play a role in a later processing stage, considering that the comparison and same-different task were exactly the same in all experimental parameters.

The effect of distance on the mean amplitudes of the early components is similar in the comparison and same-different task and might reflect early sensory processing of numerosities. In contrast, the neural task difficulty effect and the later P3 amplitude effects provide evidence for a difference between the comparison and the same-different task. In addition, the P3 component was situated at a late stadium (380–470 ms). If the P3 specifically referred to number processing, we would expect an effect of distance on the P3 component for both the comparison and the same-different task, in accordance with the similarity of the effects in both tasks on the early components (N1-P2p transition and P2p). It therefore appears likely that the effect on the P3 component for comparison reflects this additional stage of decisional processing that was proposed in previous studies (Verguts et al., 2005; Cohen Kadosh et al., 2008; Van Opstal and Verguts, 2011).

## Conclusion

To conclude, the similarity of the behavioral distance effects and the similar effects of distance on the early neural components in the comparison and same-different task suggest that both tasks are comparable. However, the difference between both tasks in task difficulty (both behavioral as neural on the TO electrode groups) and the neural results with respect to the P3 component suggest that the comparison and the same-different task differ in a later stage of processing. Namely, the effects of the P3 component are indicative of an additional decisional stage of processing for the comparison task. The observed similarities between both tasks do not necessarily contrast with the expectations of the decisional mechanisms view: this view leaves room for some shared characteristics between the CDE and the SDDE. More concrete, the decisional mechanisms view states that while representational overlap might not be a *necessary* prerequisite to explain the emergence of the CDE, it can still play a role. This might be the reason that we still observe striking similarities between both distance effects: they are both the result of representational overlap, but the results suggest that the CDE may be additionally also influenced by decisional mechanisms on top of the influence of representational overlap.

### Conflict of interest statement

The authors declare that the research was conducted in the absence of any commercial or financial relationships that could be construed as a potential conflict of interest.
